# Overview of multifunctional Tregs in cardiovascular disease: From insights into cellular functions to clinical implications

**DOI:** 10.1096/fj.202400839R

**Published:** 2024-07-09

**Authors:** Vipin Kumar, Megumi Narisawa, Xian Wu Cheng

**Affiliations:** ^1^ Department of Cardiology and Hypertension, Jilin Provincial Key Laboratory of Stress and Cardiovascular Disease Yanbian University Hospital Yanji Jilin P.R. China; ^2^ Department of Cardiology Nagoya University Graduate School of Medicine Nagoya Japan

**Keywords:** atherosclerosis, cardiovascular disease, immune disorder, inflammation, regulatory T cells

## Abstract

Regulatory T cells (Tregs) are crucial in regulating T‐cell‐mediated immune responses. Numerous studies have shown that dysfunction or decreased numbers of Tregs may be involved in inflammatory cardiovascular diseases (CVDs) such as atherosclerosis, hypertension, myocardial infarction, myocarditis, cardiomyopathy, valvular heart diseases, heart failure, and abdominal aortic aneurysm. Tregs can help to ameliorate CVDs by suppressing excessive inflammation through various mechanisms, including inhibition of T cells and B cells, inhibition of macrophage‐induced inflammation, inhibition of dendritic cells and foam cell formation, and induction of anti‐inflammatory macrophages. Enhancing or restoring the immunosuppressive activity of Tregs may thus serve as a fundamental immunotherapy to treat hypertension and CVDs. However, the precise molecular mechanisms underlying the Tregs‐induced protection against hypertension and CVDs remain to be investigated. This review focuses on recent advances in our understanding of Tregs subsets and function in CVDs. In addition, we discuss promising strategies for using Tregs through various pharmacological approaches to treat hypertension and CVDs.

## Graphical abstract

Mechanisms of Tregs protection in atherosclerosis. Tregs suppress innate and adaptive cell activation and promote SMC collagen synthesis by secreting anti‐inflammatory cytokines. Tregs facilitate the switch of M1 macrophages to M2 macrophages. Tregs inhibit T‐cell differentiation into Th1 and Th17 subtypes. Tregs suppress B cell maturation, antibody production, and immunoglobulin class switching and modulate B cell survival by secreting granzymes and perforin. Tregs inhibit DC function and maturation, induce immune tolerance of APCs, and suppress naive T‐cell activation.

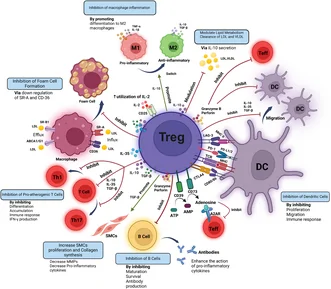

**Abbreviations**
AAA: abdominal aortic aneurysm
AMI: acute myocardial infarction
CVD: cardiovascular disease
CTLA‐4: cytotoxic T‐lymphocyte‐associated antigen‐4
DC: dendritic cells
DCM: dilated cardiomyopathy
Foxp3: forkhead box P3
GRK‐2: G protein‐coupled receptor kinase 2
HF: heart failure
IFN‐ɣ: interferon‐gamma
iTreg: inducible regulatory T cell
LAG‐3: lymphocyte activation gene‐3
LAP: latency‐associated peptide
LDL: low‐density lipoprotein
MHC‐II: major histocompatibility complex class II
nTreg: natural regulatory T cell
Ox‐LDL: oxidized low‐density lipoprotein
PD‐1: programmed cell death protein 1
PD‐L1: programmed death‐ligand 1
RHD: rheumatic heart disease
SMC: smooth muscle cell
Th: T helper
TGF‐β: transforming growth factor‐beta
Tr1: type 1 regulatory T cell
Tregs: regulatory T cells
VLDL: very low‐density lipoprotein

## 1 INTRODUCTION

Atherosclerosis is a main contributor to the development of cardiovascular diseases (CVDs), a leading cause of central vascular disease and coronary heart disease in developed countries.^1^ The significant risk in atherosclerotic CVDs is the potential rupture of unstable plaques and the development of thrombus, which can lead to severe cardiovascular conditions such as acute coronary syndrome and stroke.^2^ Adaptive and innate immune cells both contribute to the development and progression of atherosclerotic CVDs.^3^ After antigen presentation by antigen‐presenting cells, CD4 T cells undergo differentiation to form functionally distinct T helper (Th) subsets (Th1, Th2, and Th17) and regulatory T cells (Tregs).^4^ Animal studies have demonstrated that Th1 cells play a significant role in the development of atherosclerosis; however, the function of Th2 and Th17 cells is still up for debate.^5^ It is well‐known that the development and maturation of Tregs can be achieved both within the thymus and in the periphery. Tregs that are generated within the thymus are named natural Tregs (nTregs), and they exist in the absence of antigenic stimulation. The most important role of Tregs is to induce and maintain immune tolerance. The Tregs that develop and mature in the periphery are named inducible Tregs (iTregs), and they exist in the absence of pathogen stimulation.^6^ Tregs have also been found within atherosclerotic plaques, and various subsets of Tregs have been shown to have different functions, such as maintaining immunological tolerance, suppressing the excessive immune activity of effector T (Teff), and reducing the progression of atherosclerosis by inhibiting activated T‐cell responses.^7^ Tregs secrete soluble mediators, including IL‐10, IL‐35, and TGF‐β, which possess anti‐inflammatory properties.^8^ In this review, we will summarize the major role and mechanisms of Tregs in the prevention and treatment of CVDs, including myocardial infarction, myocarditis, cardiomyopathy, valvular heart diseases, heart failure, abdominal aortic aneurysm, and hypertension. The research progress and clinical potential of targeted Tregs therapy for CVDs are also discussed.

## 2 BIOLOGY OF TREGS

### 2.1 Identification of Tregs with surface markers

Studies in the 1980s and 1990s showed that mice and rats have self‐reactive T cells, as well as T cells that can suppress the autoimmunity induced by self‐reactive T cells.^9^, ^10^ The latter suppressive cells are now known as Tregs. The surface molecule CD25 was first identified as a marker for Tregs.^11^ In 2003, Rudensky's laboratory identified the transcription factor forkhead box P3 (Foxp3) as a specific marker for Tregs.^12^ Later studies showed that Foxp3 is a specific marker for human Tregs,^13^ and the CD4^+^CD25^+^Foxp3^+^ cell population were designated Treg cells.^14^, ^15^ The identification of molecular markers has increased interest in Tregs over the last decade, and experiments exploiting these markers have shown Tregs to be a potential therapeutic option for several autoimmune diseases, including atherosclerotic CVDs.

### 2.2 Development and functions of Tregs

Tregs are a specialized subtype of T lymphocytes and consist of two major subgroups: “natural” Tregs (nTregs), which form a distinct lineage in the thymus, and “induced” Tregs (iTregs), which are derived from peripheral naive conventional T cells and can be generated in vitro.^16^, ^17^ Tregs make up approximately 5%–10% of all CD4^+^ T cells.^7^ There are several types of Tregs (i.e., CD4^+^CD25^+^ Tregs, CD8^+^CD28^+^ Tregs, IL‐10‐producing type 1 Tregs [Tr1 cells] lacking Foxp3 and CD25, CD4^+^LAP^+^ Tregs, TGF‐β‐producing Th3 cells, and Tγδ Tregs). Among these, CD4^+^CD25^+^ Tregs are the most common and play a crucial role in regulating the immune response.^18^

It has been reported that the commitment of the Tregs lineage primarily relies on the engagement of T‐cell receptors with high affinity for self‐peptide major histocompatibility complexes, as well as the activation of co‐stimulatory signals through CD28. This interaction leads to the upregulation of CD25. Additionally, IL‐2 or IL‐15 plays a necessary role for the full commitment of Tregs by inducing the expression of the Foxp3, which plays a critical role in the development and function of Tregs.^19^ A deficiency of Foxp3 results in an impairment of Tregs function, which has been implicated in the development of autoimmune diseases in both humans^20^ and rodents.^12^ During homeostasis, several mechanisms ensure the stability of Tregs by maintaining the expression of FoxP3. However, under certain inflammatory conditions, Tregs can lose Foxp3 expression, compromising their regulatory function and acquiring characteristics of Teff. Foxp3 is the master regulator, and the loss of Foxp3 in such environments induces a reprogramming that converts Tregs into Foxp3^low^CD25^low^ Tregs (called exTregs).^21^ Chronic hypoxia disrupts T regulatory cell phenotype contributing to the emergence of exTreg‐Th17 cells.^22^ These exTregs that express the pro‐inflammatory cytokines (i.e., IL‐17 and IFN‐γ), lose their suppressive function, and have the potential to become pathogenic or induce autoimmunity.^21^ This supports the notion that Tregs are enriched for self‐reactivity and that unstable Tregs are associated with the progression of autoimmune disease.

The primary function of Tregs is to contribute to the control of the body's immune tolerance by preventing activated immune reactions, maintaining immune balance, and suppressing Teff.^23^ Tregs eliminate inflammatory cells by releasing granzymes^24^ and maintain immune balance by generating immunosuppressive cytokines such as TGF‐β, IL‐35, and IL‐10.^25^ IL‐10 has been shown to inhibit the production of inflammatory cytokines such as IL‐12, decreasing the Th1 response and INF‐γ production. Moreover, IL‐10 enhances phagocytic activity, facilitating the removal of cellular debris at the sites of inflammation.^26^ IL‐35, a member of the IL‐12 cytokine family, suppresses the proliferation of Th cells and promotes converting naive T cells into highly suppressive Tregs.^26^ TGF‐β can suppress activated macrophages, T and B lymphocytes, and NK cells. It was reported that both TGF‐β and IL‐10 produce a beneficial effect on Major histocompatibility II expression on antigen‐presenting cells.^18^ Additionally, Tregs can suppress the activation of Teff through a mechanism that involves the direct inhibition of CD80 and CD86 expression on the surface of antigen‐presenting cells through the action of cytotoxic T‐lymphocyte‐associated antigen‐4 (CTLA‐4).^27^ CD8^+^CD28^+^ Tregs suppress the activation of both naive and Teff as well as antibody production.^28^ Likewise, Tγδ Tregs exhibit a similar role as Tr1 cells in suppressing the growth and release of pro‐inflammatory cytokines by naive T cells and maintaining mucosal immune tolerance. Furthermore, these cells also play a role in regulating immunity against tumors and autoimmune conditions.^29^ The primary function of Th3 is to promote oral tolerance and inhibit the growth of Th1 and Th2 cells.^17^ CD4^+^LAP (latency‐associated peptide)^+^ Tregs have been found to be participated in atherosclerosis in a diet‐induced apolipoprotein E knockout (ApoE^−/−^) mouse model.^30^ Collectively, All Tregs types play a role in immune regulation and protect against atherosclerosis. This primarily entails the production of immunosuppressive enzymes, secretion of inhibitory cytokines, prevention of cell‐to‐cell interaction, disruption of metabolism, and inhibition of dendritic cell (DC) proliferation and function.^31^

Tregs express certain distinctive molecules that are crucial for their function. These molecules include FoxP3, CTLA‐4, inducible co‐stimulator, lymphocyte activation gene‐3 (LAG‐3), CD25, and CD80/CD86.^32^, ^33^ The roles of FoxP3 were described in detail as above. CTLA‐4 has been shown to play a role in controlling the suppressive functions of Tregs.^34^ Inducible co‐stimulator is necessary for precise identification by Tregs, and its deficiency impedes both immunosuppressive activity and the quantity of Tregs.^35^ Co‐stimulatory molecules, such as CD28 and CD80/CD86, are essential for homeostasis and development of Tregs.^18^, ^33^ The presence of CD25 is required for the survival of Tregs.^29^ Extensive evidence strongly indicates that impaired Tregs proliferation or malfunction leads to altered immune responses and various diseases, including autoimmune diseases, infections, tumors, and other immune‐related disorders.^36^, ^37^

## 3 ANTI‐ATHEROGENIC MECHANISMS OF TREGS IN CVDS

Several of the mechanisms by which Tregs achieve their suppressive functions and contribute to the maintenance of immune homeostasis have been identified. These mechanisms are listed in Table 1 and illustrated in Figure 1.

**TABLE 1:** Mechanisms of Tregs protection in atherosclerosis.

Role	Mechanism mediated by Tregs	Effect
Suppression of T effector cells	Tregs inhibit the differentiation of T cells into Th1 and Th17 subtypes. Tregs effectively suppress immune responses associated with Th1 and Th17 in atherosclerosis through the secretion of IL‐10, IL‐35, and TGF‐β.^30^, ^38^	Decreased activation, proliferation, and progression to apoptosis in pro‐atherogenic T cells
Inhibition of macrophage‐induced inflammation	Tregs facilitate the conversion of M1 macrophages to M2 macrophages through the release of IL‐10, which has a positive effect on preventing the development of atherosclerosis.^39^ The activation of M2 macrophages enhances the stability of plaques by stimulating collagen synthesis and SMC proliferation.^40^	Decreased inflammation and the production of pro‐inflammatory cytokines Improvement in the stability of atherosclerotic plaques
Modulation of dendritic cells (DCs) development and function	Tregs regulate dendritic cell maturation and function via cytokines such as IL‐10 and TGF‐β and surface molecules including PDL‐1/2, CTLA‐4, and LAG‐3.^27^, ^41^, ^42^	Inhibition of dendritic co‐stimulatory molecules leads to a reduction in the activation of effector cells
Control of lipid metabolism and foam cell formation	Tregs suppress lipid accumulation in peritoneal macrophages by reducing the activity of scavenger receptor class A and CD36. This mechanism effectively prevents foam cell formation.^43^	Inhibition of foam cell formation
Regulation of pro‐inflammatory B cells	Tregs regulate B cell survival by releasing granzymes and perforin.^44^	Reduced atherosclerosis by inhibition of effector T‐cell activation

Abbreviations: CD, clusters of differentiation; CTLA‐4, cytotoxic T‐lymphocyte‐associated antigen‐4; IL, interleukin; LAG‐3, lymphocyte activation gene‐3; M1/2, macrophage 1/2; PDL‐1/2, programmed death‐ligand 1/2; SMCs, smooth muscle cells; TGF‐β, transforming growth factor‐beta; Th, T helper.

**FIGURE 1:**
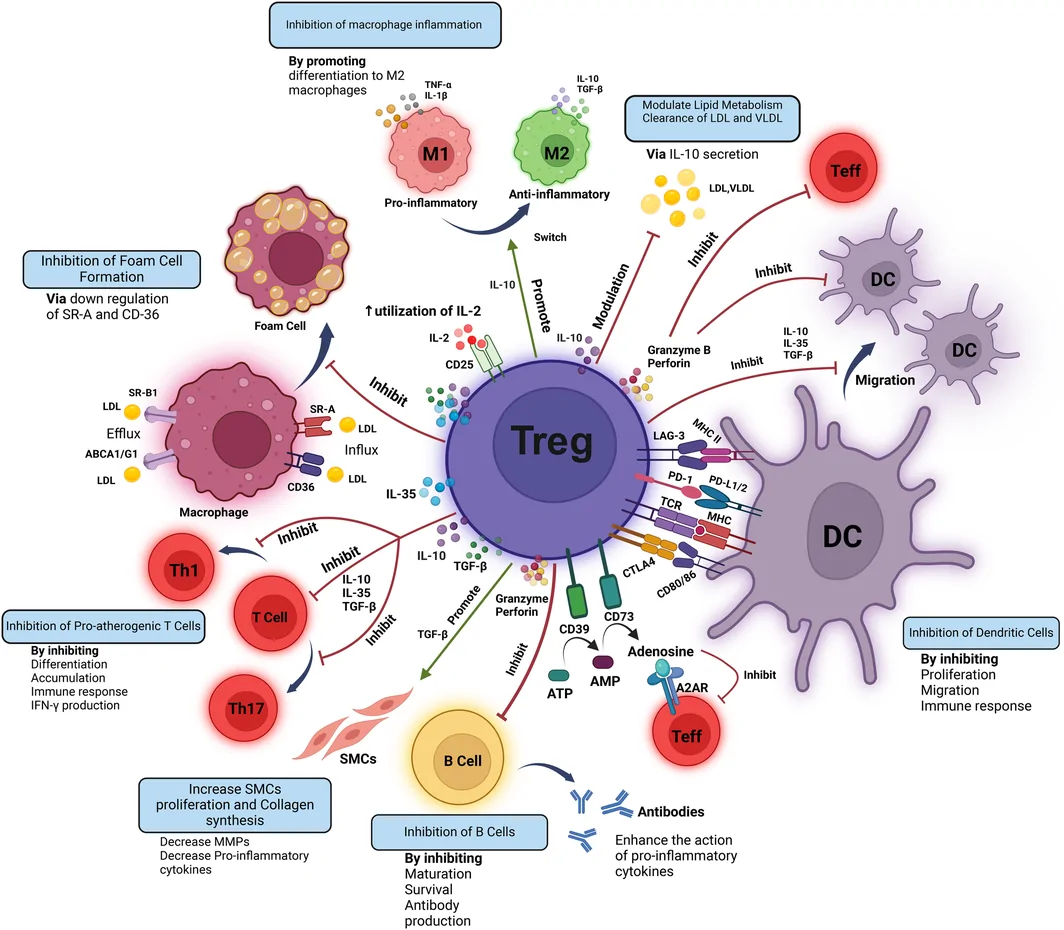
Mechanisms of Tregs protection in atherosclerosis. The mechanism of Tregs in protection against atherosclerosis. By secreting IL‐10, IL‐35, and TGF‐β, Tregs reduce inflammatory cell recruitment and activation, inhibit effector T‐cell differentiation and inflammation, and promote SMC survival and proliferation and collagen synthesis. Tregs facilitate the switch of M1 macrophages to M2 macrophages through the release of IL‐10. Tregs inhibit T‐cell differentiation into Th1 and Th17 subtypes and suppress immune and inflammatory responses by inhibiting the production of the pro‐inflammatory cytokine IFN‐γ. Tregs also suppress B cell maturation, antibody production, and immunoglobulin class switching and modulate B cell survival by secreting granzymes and perforin. Tregs inhibit DC function and maturation, induce immune tolerance of APCs, and suppress naive T‐cell activation. Tregs improve plaque stability by inhibiting monocyte recruitment and promoting SMC proliferation. In addition, Tregs inhibit lipid and foam cell formation by decreasing the expression of CD36 and SR‐A and B1 reducing plasma VLDL, LDL, and cholesterol levels. A2AR, adenosine receptor; ABCA1/G1, ATP‐binding cassette transporters A1/G1; AMP, adenosine monophosphate; APC, antigen presented cell; ATP, adenosine triphosphate; CD, clusters of differentiation; CTLA‐4, cytotoxic T‐lymphocyte‐associated antigen‐4; DC, dendritic cell; IFN‐γ, interferon‐gamma; IL, interleukin; LAG‐3, lymphocyte activation gene‐3; LDL, low‐density lipoprotein; M1/2, macrophage 1/2; MHC, major histocompatibility; PD‐1/PD‐L1, programed cell death protein 1/programmed death‐ligand 1; SMCs, smooth muscle cells; SR‐A/B1, scavenger receptor class A/B1; TCR, T‐cell receptor; Teff, T effector; TGF‐β, transforming growth factor‐beta; Th, T helper; TNF‐α, tumor necrosis factor alpha; Tregs, regulatory T cells; VLDL, very low‐density lipoprotein.

### 3.1 Suppression of effector T and B cell activities

Atherosclerosis is a complex process that involves intricate interactions among various immune cells and cytokine networks. The majority of the atherogenic T cells are effector Th1 and Th17 cells.^45^ Several studies have shown that Th1 cells play a role in promoting the infiltration of monocytes and T cells into atherosclerotic plaques. They also stimulate antigen‐presenting cells within the lesions by releasing IFN‐γ, which further hastens the development of atherosclerosis and enhances plaque vulnerability.^46^ Th17 plays a crucial role in the development of atherosclerosis by releasing chemokines and the pro‐inflammatory cytokines IL‐17 and IL‐6.^47^ Tregs inhibit the differentiation of T cells into Th1 and Th17 subtypes. Notably, Tregs effectively suppress immune responses associated with Th1 and Th17 in atherosclerosis through the secretion of TGF‐β, IL‐10, and IL‐35. IL‐10 is produced by Treg1 cells, which contribute to the reduction in atherosclerotic plaques by inhibiting Th1 differentiation and inhibiting macrophage and T‐cell accumulation and cytokine production in atherosclerotic plaques.^30^, ^38^ It has been reported that IL‐10 deficiency in C57BL/6 mice fed an atherogenic diet resulted in the early formation of atherosclerotic lesions with increased populations of activated T cells and other inflammatory cells, and increased the production of pro‐inflammatory cytokines. In the same study, overexpression of IL‐10 by T cells resulted in a reduction of atherosclerosis in low‐density lipoprotein receptor (Ldlr)^−/−^ mice.^48^ TGF‐β attenuates atherosclerosis by preventing the migration and inflammatory cell activation in atherosclerotic plaques and increases plaque stability by promoting the survival and proliferation of vascular smooth muscle cells (SMCs) and collagen biosynthesis.^29^ Blocking TGF‐β signaling can lead to a pro‐inflammatory state within the vessel wall and the formation of unstable atherosclerotic plaques.^49^ The overexpression of TGF‐β in the hearts of ApoE^−/−^ mice results in reduced lesion size, decreased T‐cell infiltration, and increased collagen production in plaques, demonstrating that TGF‐β plays a role in the production of vascular SMCs matrix and the stability of plaques in atherosclerosis.^50^, ^51^ A novel cytokine, IL‐35, comprises two subunits: Epstein–Barr virus‐induced gene3 and IL‐12 p35. Notably, both of these subunits are highly co‐expressed in atherosclerotic lesions.^52^ In ApoE^−/−^ mice, a deficiency of IL‐12p35 leads to a decrease in IL‐35 levels, inhibiting the development and function of Tregs. This imbalance between Th17 and Tregs exacerbates atherosclerosis but stabilizes the plaque.^53^ In animal studies, the use of recombinant human IL‐35 elevates the levels of Tregs in the circulating and local Tregs while decreasing plaque size in ApoE^−/−^ mice. This suggests that IL‐35 attenuates atherosclerosis by enhancing the Tregs immune response.^54^ When ApoE^−/−^ mice were fed a high‐fat diet and immunized intraperitoneally for 6 weeks, they developed antibodies against IL‐2. The presence of these antibodies increased plaque size. Notably, anti‐IL‐2 therapy exhibited a significant anti‐atherosclerotic effect in this model.^55^ Other authors reported that administration of IL‐2/anti‐IL‐2 mAb to ApoE^−/−^ mice resulted in an increase in Tregs, a reduction in inflammation, and mitigation of both plaque development and progression.^56^

Unlike T cells, only a limited number of B cells can be found within the atherosclerotic plaques.^57^ However, B2 cells produce antibodies that readily bind to atherosclerotic plaques; they can exacerbate atherogenesis by enhancing the action of pro‐inflammatory cytokines.^58^ IgE potentially elevates plasma low‐density lipoprotein (LDL) levels and polarizes macrophages toward a pro‐inflammatory M1, enhancing the risk of atherosclerosis.^59^ Transferring B2 cells into ApoE^−/−^ mice accelerated atherosclerosis progression.^60^ Moreover, Tregs regulate B‐cell survival by releasing granzymes and perforin.^44^ In Ldlr^−/−^ and ApoE^−/−^ mice, the administration of anti‐CD20 antibody resulted in B cell depletion, which effectively reduced atherosclerosis by inhibiting Teff cell activation.^61^ Therefore, the regulation of B cell functions represents a novel mechanism through which Tregs protect against atherosclerosis.

### 3.2 Inhibition of macrophage‐induced inflammation

Macrophages can be divided into two types according to their polarization phenotype: classically activated M1 macrophages and activated M2 macrophages.^62^ Macrophages polarize into the M1 type upon stimulation with lipopolysaccharides or IFN‐γ, which secretes TNF‐α, IL‐1β, and other inflammatory mediators that contribute to the initiation and progression of atherosclerosis.^63^ M2 macrophages undergo polarization in response to the cytokines IL‐4 and IL‐13. They secrete anti‐inflammatory factors such as IL‐10, TGF‐β, and vascular endothelial growth factor, which have anti‐atherosclerotic effects and stabilize plaques.^64^ Tregs facilitate the switching of M1 macrophages to M2 macrophages through the release of IL‐10, which has a positive effect on preventing the development of atherosclerosis.^39^ Tregs and monocytes cultured together promote the development of the M2 phenotype. Additionally, when monocytes treated with Tregs were stimulated with lipopolysaccharide, their production of pro‐inflammatory cytokines noticeably decreased.^65^ Tregs play a role in improving macrophage efferocytosis through the secretion of IL‐13, which then stimulates the production of IL‐10 in macrophages.^66^ The activation of M2 macrophages enhances the stability of plaques by stimulating collagen synthesis and vascular SMC proliferation.^40^ This indicates that Tregs contribute to the stability of lesions by promoting the development of M2 macrophages. Tregs decrease the likelihood of plaque rupture by increasing the presence of SMCs and collagen. At the same time, Tregs also reduce the secretion of pro‐inflammatory cytokines and matrix metalloproteinases in atherosclerotic lesions in ApoE^−/−^ mice.^67^

### 3.3 Modulation of DC development and functions

DCs are the cell type primarily responsible for initiating antigen‐specific immunity and tolerance.^68^ DCs induce the maturation and polarization of naive T cells by presenting antigenic peptides on a major histocompatibility complex class II (MHC‐II) molecule. They also express the relevant membrane‐bound signaling molecules and release cytokines.^69^ Tregs have the ability to inhibit DCs through various mechanisms. In addition to secreting three main immunosuppressive cytokines (TGF‐β, IL‐10, and IL‐35), Tregs also express certain cell surface molecules including CTLA‐4^27^ and LAG‐3.^41^ CTLA‐4 on Tregs interacts with CD80/CD86 on DCs, thereby inhibiting the ability of DCs to activate naive T cells.^42^ LAG‐3 interacts with MHC‐II on the surface of DCs, resulting in inhibition of DCs maturation and immunostimulatory function.^70^ Overexpression of CTLA‐4 has been correlated with an increased presence of Tregs and a reduction in atherosclerosis.^71^ Furthermore, the results from hypercholesterolemic Ldlr^−/−^ mice and cancer patients showed that PD‐1 in combination with death protein 1 (PD‐L1) or programmed death‐ligand 1 (PD‐L1) confers protection against atherosclerosis.^72^, ^73^ The lack of PD‐1/PD‐L1 leads to increased infiltration and influence of CD4^+^ and CD8^+^ T cells within the plaque, resulting in the escalation and worsening of atherosclerotic plaque formation in hyperlipidemic Ldlr^−/−^ mice.^72^ Further research on the precise molecular and cellular mechanisms associated with Treg‐mediated DC modulation is required to harness the full potential of this knowledge for clinical applications.

### 3.4 Control of lipid metabolism and foam cell formation

A significant contributor to atherosclerosis is dyslipidemia, which is characterized by elevated levels of LDL and reduced levels of high‐density lipoprotein. A specific type of modified LDL, known as oxidized low‐density lipoprotein (ox‐LDL), plays a role in the development of atherosclerotic plaques.^74^ The impaired uptake of ox‐LDL by lectin‐like oxidized Ldlr‐1, class A1 scavenger receptors, CD36, and impaired cholesterol efflux by adenosine triphosphate‐binding cassette transporter‐A1 and adenosine triphosphate‐binding cassette transporter‐G1 lead to cholesterol deposition and consequently induce vascular macrophages and SMCs to become foam cells.^75^, ^76^ Therefore, regulating the influx and efflux of cholesterol in macrophages is crucial to effectively prevent atherosclerosis.^77^ Numerous studies have demonstrated that enhancing the expression of cholesterol transporter‐related protein adenosine triphosphate‐binding cassette transporter‐A1/G1 and scavenger receptor class‐B1 and the efflux of cholesterol in macrophages can prevent the development of atherosclerosis.^78^, ^79^ Studies have shown that Tregs suppress lipid deposition in peritoneal macrophages by reducing the activity of scavenger receptor class‐A and CD36. This mechanism effectively prevents foam cell formation.^43^ The recruitment of bone marrow‐derived mononuclear cells into the circulation is facilitated by monocyte chemotactic factor‐1. Subramanian et al. found that Tregs can inhibit monocyte infiltration into plaques by suppressing monocyte chemotactic factor‐1 expression. This inhibition also has a detrimental effect on the development and accumulation of macrophage‐derived foam cells.^80^ Furthermore, IL‐10 decreases LDL and very low‐density lipoprotein (VLDL) levels in Ldlr^−/−^ mice. In addition, Tregs depletion mice have elevated plasma cholesterol and VLDL levels (Ref.).^81^ Thus, Tregs can produce a vascular benefit through the modulation of lipid metabolism and foam cell formation.

### 3.5 Other mechanisms

Tregs exert their suppressive function by disrupting the metabolism of target cells. CD25 is expressed at high levels on the surface of Tregs, which promotes higher utilization of IL‐2. The uptake of IL‐2 by Tregs has been suggested to suppress the growth of Teff and induce metabolic disturbances leading to their death and immunosuppression. This is due to IL‐2 playing a vital role in the growth and stimulation of Teff.^82^ Additionally, Tregs express high levels of CD39 and CD73 and play a substantial role in immunosuppression. They are capable of hydrolyzing adenosine triphosphate released from damaged/dying cells into adenosine, which acts as an anti‐inflammatory substance in numerous compromised blood vessels, such as atherosclerotic coronary arteries.^83^ This adenosine then inhibits the activity of Teff by attaching to the adenosine A2A receptor. Activation of the adenosine A2A receptor not only inhibits the activity of Teff, but also promotes the Tregs immunosuppressive activity.^84^, ^85^ Finally, an additional potential mechanism for the suppressive function of Tregs involves cytolysis, mainly through granzyme B and perforin. This mechanism is crucial for Treg‐mediated suppression of tumor clearance.^86^, ^87^ Further studies are needed to determine how cell contact‐dependent and humoral factor‐mediated Treg‐suppression mechanisms effectively control immune responses depending on the type of responder T cells (their T‐cell receptors affinity and activation state) or immune cells (DCs, B cells, or other cells) and the phase, location, and type of inflammation.

## 4 TREGS IN CVDS

There is increasing evidence of a significant role of Tregs in the development and progression of CVDs (Figure 2).

**FIGURE 2:**
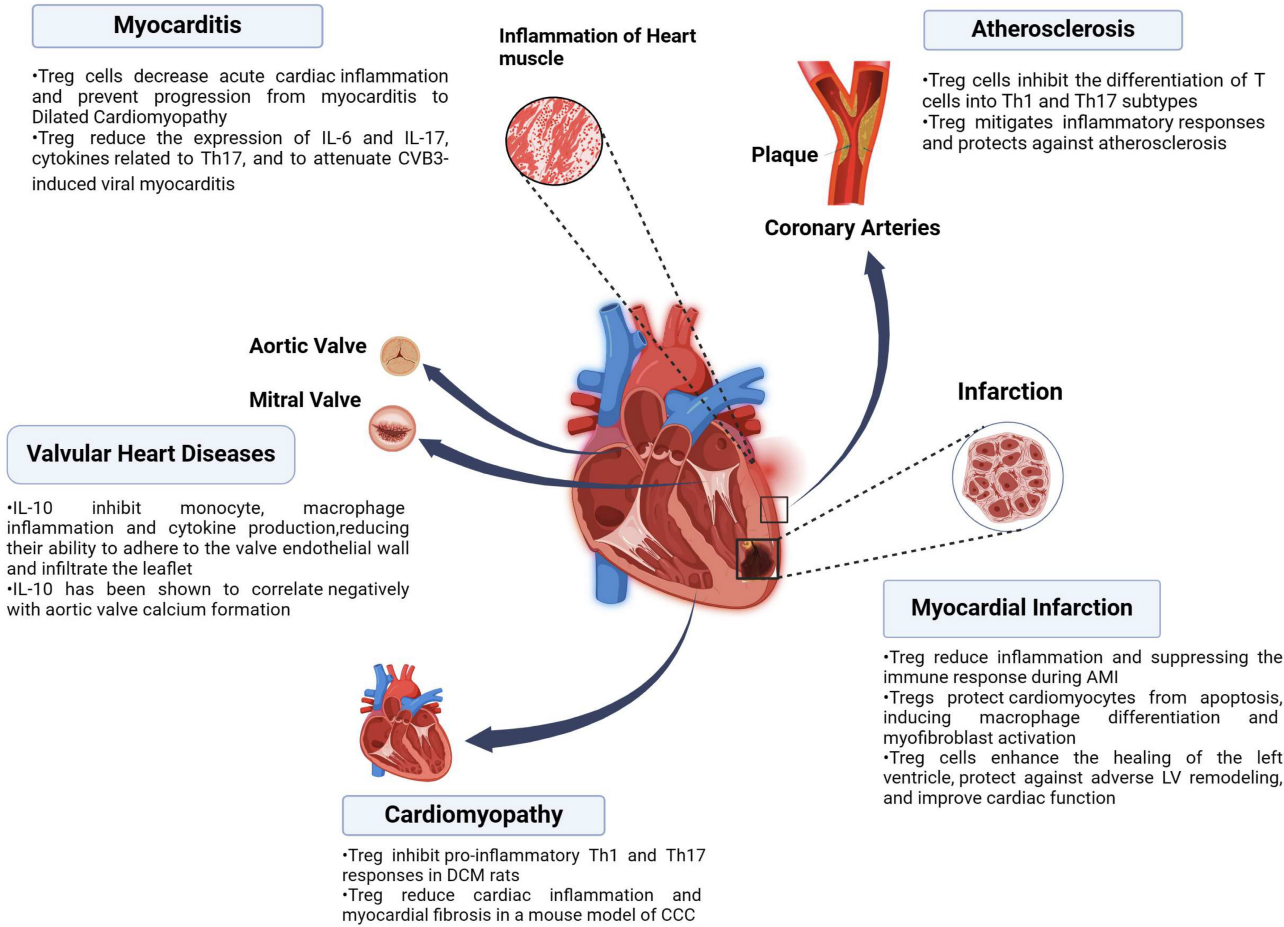
Tregs in cardiovascular diseases. Tregs play a critical role in modulating immune responses and inflammation in cardiovascular disease. Tregs reduce acute cardiac inflammation in myocarditis, preventing progression to dilated cardiomyopathy (DCM), and attenuate Coxsackievirus B3‐induced viral myocarditis by reducing IL‐6 and IL‐17 expression. In atherosclerosis, Tregs inhibit T‐cell differentiation into pro‐inflammatory Th1 and Th17 subtypes, attenuating inflammatory responses and protecting against disease progression. In valvular heart disease, Treg‐produced IL‐10 inhibits monocyte and macrophage inflammation, reduces adherence to valve endothelial walls, and negatively correlates with aortic valve calcification. In cardiomyopathy models, Tregs inhibit pro‐inflammatory Th1 and Th17 responses, reducing cardiac inflammation and myocardial fibrosis. During myocardial infarction, Tregs reduce inflammation, protect cardiomyocytes from apoptosis, induce macrophage differentiation and myofibroblast activation, enhance left ventricular healing, protect against adverse remodeling, and improve cardiac function. These protective roles make Tregs a potential therapeutic target for treating cardiovascular diseases. AMI, acute myocardial infarction; CCC, chronic Chagas cardiomyopathy; CVB3, coxsackievirus B3; DCM, dilated cardiomyopathy; IL, interleukin; LV, left ventricle; Th, T helper; Tregs, regulatory T cells.

### 4.1 Hypertension

Globally, hypertension is the most important modifiable risk factor for CVDs and mortality from all causes.^88^ Inflammation is involved in many pathological processes leading to elevated blood pressure. The immune response is crucial in initiating and advancing hypertension and end‐organ damage.^89^ Treg deficiency promotes the development of hypertension by increasing both innate and adaptive immune responses.^90^ Reducing the number of Tregs significantly increases systolic blood pressure.^91^ Legumain, also known as asparagine endopeptidase, belongs to the C13 family of cysteine proteases.^92^ Its activity in human CD4^+^ T cells promotes the induction of Th1 and increases the production of IFN‐γ.^93^ Legumain is also present in human Tregs, and its inhibition can improve the function of Tregs in CD4^+^ T cells from both humans and mice.^94^, ^95^ One study reported that legumain inhibition attenuated Ang‐II or deoxycorticosterone acetate/salt‐induced hypertension and reduced target organ damage in mice, in association with decreases in T‐cell infiltration and local inflammation in the aorta and kidney due to increased Tregs numbers.^96^ However, inducing Tregs by adoptive transfer has been shown to ameliorate hypertension, attenuate vasodilator impairment and immune cell recruitment, and suppress oxidative stress, autophagy, and inflammation. Together, these effects improve the function of the hypertensive microvasculature.^97^, ^98^ Furthermore, the combined absence of complement C3a receptor and complement C5a receptor was shown to modulate Tregs activity in a mouse model of hypertension, and this modulation in turn attenuated angiotensin II‐induced expression of inflammatory cytokines, minimized target organ damage, and reduced increased blood pressure.^99^ Activation of liver kinase B1 by hydrogen generated from cystathionine γ‐lyase promotes the differentiation and proliferation of Tregs, leading to a reduction in immune inflammation in the blood vessels and kidneys, and ultimately conferring protection against the development of hypertension.^97^ In a cohort of patients with pregnancy‐induced hypertension, activation of the programmed cell PD‐1/PD‐L1 pathway significantly increased the Tregs ratio and Foxp3 mRNA expression, and increased the levels of anti‐inflammatory factors such as TGF‐β, IL‐10, and IL‐35; together, these effects ameliorated the gestational hypertension.^100^ In contrast, increasing the proportion of functional Tregs and supplementing anti‐inflammatory cytokines or reducing pro‐inflammatory cytokine levels may be new approaches for the treatment of hypertension.

### 4.2 Atherosclerosis

Experimental and clinical studies have shown that innate and adaptive immune responses are critical in inducing vascular inflammation and atherosclerosis.^101^ Tregs are an important subset of T cells that offer defense against autoimmunity and could be used to treat autoimmune‐like conditions, such as atherosclerosis.^23^ The study results indicate that Tregs numbers increase within regressing plaques in several mouse models of atherosclerosis regression.^39^ Single‐cell RNA sequencing analyses revealed that the Tregs profile changes during lipid‐lowering‐induced atherosclerotic plaque regression.^39^ Tregs from regressing plaques are derived from peripheral differentiation of naive T cells. In contrast, Tregs from progressive plaques are derived from the thymus and show increased activation and metabolic activity.^39^ The researchers also show that Tregs are essential for the regression of atherosclerosis. Depletion of Tregs in mice blocked the regression of atherosclerosis achieved with aggressive lipid‐lowering therapy. In addition, reductions in Tregs populations have been shown to be harmful in atherosclerotic processes in animal and human studies; for example, ApoE^−/−^ mice fed a hypercholesterolemic diet show decreased numbers of Tregs and increased atherosclerosis compared with mice fed a normal diet.^102^ In another mouse model of atherosclerosis using Lldr^−/−^ mice, diet‐induced hypercholesterolemia initially led to an increase in circulating and plaque Tregs. However, their numbers were found to decrease in the later stages of the disease.^103^ Several studies have shown that acute coronary syndrome patients have lower numbers of circulating Tregs compared with those with stable angina or normal coronary arteries.^104^, ^105^ Furthermore, ox‐LDL affects the Treg/Th17 ratio by inducing Tregs apoptosis via the Fas/FasL pathway and promoting NF‐κB‐associated Th17 proliferation.^106^ Imbalances between Tregs and Th17 cells have been significantly associated with both atherosclerosis and acute coronary syndrome.^107^ Depletion of CD4^+^CD25^+^ Tregs using anti‐CD25 antibodies accelerates atherosclerotic lesion development and increases plaque vulnerability in ApoE^−/−^ mice.^108^ Atherosclerosis is also increased in LDLR^−/−^ mice reconstituted with bone marrow depleted of FOXP3^+^ Tregs using a diphtheria toxin‐based procedure.^81^ The adoptive transfer of Tregs mitigates inflammation and protects from atherosclerosis.^108^, ^109^ These findings highlight a relationship between Tregs numbers, their regulatory role in the immune system, and the progression of atherosclerosis. Increasing or restoring the number of Tregs, along with their immunosuppressive properties, may serve as a fundamental immunotherapy approach to treating atherosclerosis.

### 4.3 Myocardial infarction

Studies have shown that the inflammatory response triggered by acute myocardial infarction (AMI) is a significant contributor to myocardial injury.^110^ Recent research suggests that Tregs, identified by the presence of CD4^+^CD25^+^FOXP3^+^, may play an important role in reducing inflammation and suppressing the immune response during AMI. Tregs appear to accomplish these effects by the release of anti‐inflammatory cytokines such as TGF‐β and IL‐10, as well as by their interaction with other cell types through direct contact.^111^

Laboratory studies have shown that mice lacking Tregs have larger infarcts and more adverse left ventricular remodeling. Conversely, the induction of Tregs has been shown to attenuate infarct size and protect myocardial function in mouse models of myocardial infarction.^112^, ^113^ In a mouse model of AMI, Tregs played a role in enhancing macrophage polarization to the M2 type, leading to improved immune homeostasis and cardiac repair. On the contrary, in AMI model mice with depleted Tregs, macrophages were polarized to the M1 type, resulting in an enhanced inflammatory response.^114^ In support of these findings, another study reported that activation of Tregs by dendritic cell‐derived exosomes resulted in polarization of macrophages to the M2 type. This resulted in a significant increase in the infiltration of Tregs and M2 macrophages in the border zone of AMI model mice, leading to an improvement in cardiac function.^115^ As an inflammatory inducer of chemokine receptor‐2^+^ macrophages and DCs, chemokine ligand‐17 inhibits Tregs infiltration by preferentially activating chemokine receptor‐4. However, the elimination of the chemokine ligand‐17 gene resulted in increased Treg infiltration, decreased gene expression, and improvement in cardiac function and remodeling in AMI mice.^116^ After myocardial infarction, C‐X‐C Motif chemokine ligand‐12/C‐X‐C motif chemokine receptor‐4 attracts inflammatory cells to the infarct zone. A C‐X‐C motif chemokine receptor‐4 antagonist specifically augmented the recruitment of splenic Tregs to the infarct zone by inducing DCs, suppressed pro‐inflammatory macrophage gene expression, improved cardiac function, and promoted cardiac repair in AMI‐reperfused mice.^117^ In addition, CD73 from CD4^+^ Foxp3^+^ Tregs can bind to CD4^+^ Foxp3^−^ Teff and lower levels of IFN‐γ, TNF‐α, IL‐1β, and IL‐17. This suppresses inflammation and protects against AMI.^118^ Tregs can regulate the inflammatory response and facilitate tissue repair after an AMI. Treg‐based therapies have the potential to improve treatment and recovery for AMI patients. Continued clinical studies and researches are essential to explore the therapeutic benefits of Tregs in this context.

### 4.4 Myocarditis

Myocarditis is an inflammatory condition that affects myocardial cells. It presents various clinical and histological manifestations of immune‐related cardiac pathology, potentially resulting in acute heart failure, sudden cardiac death, or chronic dilated cardiomyopathy.^119^, ^120^ In patients with myocarditis, Tregs decrease acute cardiac inflammation and protect against progression of myocarditis to dilated cardiomyopathy (DCM).^121^ Mutations in the FOXP3 gene result in the development of severe autoimmune, inflammation, and allergic reactions in various organs in both rodents and humans.^122^ The adoptive transfer of CD4^+^ T cells lacking glucocorticoid‐induced tumor necrosis factor receptor into nude mice resulted in the depletion of Tregs, ultimately leading to the spontaneous onset of fatal autoimmune myocarditis.^123^ Consistent with this finding, exacerbation of cardiac fibrosis was observed when Tregs were depleted in coxsackievirus‐B3 infected mice by administration of anti‐CD25 monoclonal antibodies.^124^ Patients with acute myocarditis showed a significant decrease in circulating Tregs compared with healthy volunteers.^125^ However, the adoptive transfer of Tregs before coxsackievirus‐B3 infection had the dual effect of reducing the inflammatory response to the virus and aiding in eliminating the virus from the mouse myocardium.^126^ Moreover, the adoptive transfer of isolated Tregs into mice after coxsackievirus‐B3 infection resulted in a significant reduction in cardiac fibrosis,^124^ and the recruitment of Tregs cells ameliorated myocarditis in a murine model of chronic Chagas cardiomyopathy.^127^ In a mouse model of experimental autoimmune myocarditis, the expansion of Tregs by adenovirus‐mediated CTLA4 Ig gene therapy reduced the disease severity.^128^ On the contrary, with respect to TGF‐β, this factor has been found to protect mice against coxsackievirus‐induced myocarditis,^129^ and to play a role in a positive feedback loop that converts naive CD4^+^ T cells into regulatory T cells.^130^ Oppositely, in the later stages of myocarditis, TGF‐β has been shown to promote disease and adverse cardiac remodeling. In experimental autoimmune myocarditis, TGF‐β‐mediated Wnt secretion leads to myofibroblast differentiation and myocardial fibrosis,^131^ while treatments targeting TGF‐β were shown to prevent fibrosis and heart failure in mice.^132^ As anticipated, the activation of Tregs by IL‐37 and the production of IL‐10 have been found to reduce the expression of IL‐6 and IL‐17, cytokines related to Th17, and to attenuate coxsackievirus‐B3 induced viral myocarditis.^133^ Similar to TGF‐β, IL‐35 has also been shown to facilitate naive T cell converting to a regulatory phenotype.^134^, ^135^ Token together, modulation of autoimmunity may be useful in the prevention of myocarditis, and modulation of Tregs abundance and function of Tregs may be a therapeutic approach to prevent these diseases.

### 4.5 Cardiomyopathy

Accumulating laboratory evidence indicates that Tregs also play a beneficial role in human DCM by inhibiting the dominant Th1 cell response and the inhibition of pro‐inflammatory cytokines such as IL‐2.^136^, ^137^ Clinical studies have reported reduced Tregs numbers and reduced suppressive function of Tregs in patients with DCM compared with the normal population,^138^, ^139^ suggesting that increasing the numbers of Tregs and enhancing their function could benefit to DCM treatment. In an in vivo study, adoptive transfer of H310A1 immune CD4CD25 cells in transgenic mice resulted in a decrease in heart weight and plasma troponin I levels, as well as an amelioration of existing DCM.^140^ Adoptive transfer of Tregs protects against cardiac fibrosis induced by coxsackievirus B3.^124^ A recent study found that administering two doses of human umbilical cord mesenchymal stromal cells increased Tregs and inhibited pro‐inflammatory Th1 and Th17 responses in DCM rats. This resulted in a decrease in cardiac fibrosis and cardiomyocyte apoptosis.^141^ In addition, immunotherapy with tolerogenic dendritic cells inhibits the progressive cardiac inflammation and myocardial fibrosis in a mouse model of chronic Chagas cardiomyopathy. This is accomplished through secretion of IL‐10 in tolerogenic dendritic cells, which promotes differentiation of Tregs and increases their immunosuppressive effect.^142^ Interestingly, treatment with human recombinant granulocyte colony‐stimulating factor increases recruitment of cardiac Tregs and reduces cardiac inflammation, fibrosis, and parasite burden in mice with chronic Chagas cardiomyopathy.^143^ On the contrary, high insulin levels can decrease the ability of Tregs to suppress inflammatory responses by regulating the protein kinase B/mammalian target of rapamycin signaling pathway.^144^ Another study has shown that Tregs were significantly reduced in diabetic cardiomyopathy mice due to hyperinsulinemia. This process was found to be G protein‐coupled receptor kinase (GRK)‐2 dependent, suggesting that increased expression of GRK‐2 in circulating lymphocytes impairs Tregs differentiation and affects immune homeostasis in diabetic cardiomyopathy mice.^145^ Paroxetine directly binds to GRK‐2 and blocks kinase activity, with 60‐fold selectivity for GRK‐2 over other GRKs and has been shown to improve cardiac function in diabetic cardiomyopathy mice.^146^ Therefore, the restoration of Tregs proportions and function may be a novel and promising therapeutic strategy for immunomodulation and treatment of CVD.

### 4.6 Valvular heart diseases

Rheumatic heart disease (RHD) is an autoimmune consequence of rheumatic fever that typically develops after Group A streptococcal throat infection.^147^ Tregs play a crucial role in RHD and are an important part of the autoimmune response. Treg deficiency has been found in patients with RHD compared with control groups.^148^ Depletion of Tregs may also be due to streptococcal antigens, as M protein acts as a potential inhibitor of Tregs activity.^149^ At the same time, an increased percentage of Th17 cell‐associated cytokines plays a crucial role in the progression of RHD.^150^ Bas et al. have reported a deficiency of Tregs in patients with rheumatic mitral valve disease and suggest that balancing the Th17/Tregs levels may have a therapeutic role in patients with RHD.^148^ Although an increase in Tregs populations has been observed in patients with severe aortic stenosis, followed by a decrease in levels of Tregs after replacement of the aortic valve,^151^ no definitive conclusions have been reached regarding the precise role of Tregs in this condition. However, evidence for Tregs cell function can be drawn from their involvement in atherosclerosis.^39^, ^45^, ^152^ Furthermore, IL‐10, secreted by Tregs, contributes to a dormant microenvironment by inhibiting monocyte and macrophage inflammation and cytokine production, thereby reducing their ability to adhere to the valve endothelial wall and infiltrate the leaflet. Circulating IL‐10 has been shown to correlate negatively with aortic valve calcium formation.^153^ The current understanding of Tregs in valvular heart disease is promising but still in its emerging stages. The ongoing research and development have the potential to identify novel therapeutic approaches that could transform the treatment of these diseases.

### 4.7 Heart failure

Heart failure (HF) is a complex and multifactorial process. Activation of the immune system has a significant impact on the progression of HF.^154^, ^155^ Studies have shown decreased circulating Tregs in patients with chronic HF, which may contribute to immune dysregulation, chronic inflammation, and progression of HF.^156^, ^157^ Th17/Tregs imbalance has also been observed in patients with HF,^158^ and in rats with left anterior descending artery ligation‐induced HF, in the latter case accompanied by significantly increased Th17 cell levels and decreased Tregs levels.^159^ Failing hearts exhibit a predominance of Th2 and Th17 cells compared with Th1 cells and Tregs.^160^ Adoptive transfer of Tregs attenuates interstitial fibrosis, myocardial matrix metalloproteinase activity, and cardiac apoptosis and reduces neutrophil, macrophage, lymphocyte infiltration, TNF‐α, and IL‐1β production.^161^ The expansion of Tregs mediated by the IL‐2/anti‐IL‐2 complex slightly improved myocardial function and attenuated myocardial fibrosis in a mouse model of myocardial ischemia/reperfusion injury.^162^ Additionally, NADPH oxidase‐2 limits the suppressive activity of Tregs, leading to increased infiltration/proliferation of Teff, including Th17 cells, and enhances Ang‐II‐induced hypertension, cardiac fibrosis, and hypertrophy, and deletion of NADPH oxidase‐2 increases cardiac Tregs.^163^ A recent study demonstrated that β‐hydroxybutyrate attenuated cardiac inflammation and remodeling, and these effects may have been related to an increase in Tregs through downregulation of the NADPH oxidase‐2/glycogen synthase kinase‐3β pathway.^164^ Collectively, these findings show that Tregs may be a significant predictor of prognosis and a potential target for the treatment of HF.

### 4.8 Abdominal aortic aneurysm

Abdominal aortic aneurysm (AAA) is characterized by progressive dilation of the aorta, often leading to a potentially fatal rupture.^165^ Experimental and clinical studies suggest that inflammation caused by the accumulation of Teff and macrophages in aneurysmal lesions contributes to the development of AAA.^166^ Patients with AAA have been reported to exhibit reduced Tregs numbers and decreased expression of Foxp3 in peripheral Tregs when compared to healthy control subjects.^167^ Another study found that Tregs in peripheral blood samples from individuals with AAA were lower than those of a control group.^168^ Tregs depletion by anti‐CD25 monoclonal antibody treatment promotes angiotensin II‐induced AAA progression and rupture in C57Bl/6 mice.^169^ Conversely, adoptive transfer of Tregs significantly prevents angiotensin II‐induced AAA in ApoE^−/−^ mice.^170^ Ex vivo expansion of human Tregs with IL‐2 results in reduced aneurysm progression in a humanized mouse model of AAA.^171^ In addition, propionate, a short‐chain fatty acid produced through the bacterial fermentation of dietary fiber in the colon, has been shown to alleviate inflammatory bowel diseases via an expansion of Tregs.^172^ Propionate expanded colonic Tregs attenuated AAA in both Ca3(PO4)2‐induced and elastase‐induced mouse models of AAA.^173^ Short‐chain fatty acid‐enriched starch can be used as a dietary supplement for high‐risk patients with AAA. Furthermore, simvastatin treatment prevented Ang‐II‐induced AAA formation in ApoE^−/−^ mice, possibly by promoting Tregs accumulation.^174^ These reports suggest that impaired immune regulation by Tregs may be involved in the development of AAA and that Tregs induction may be an attractive therapeutic approach for AAA.

## 5 TARGETING TREGS FOR THE PREVENTION AND TREATMENT OF CVDS

Evidence suggests that an imbalance of Teff and Tregs is the primary driver of atherogenesis and plaque instability. This imbalance initiates a cascade of inflammatory responses. Additionally, both innate and adaptive immune responses play a crucial role in the progression of atherosclerosis. As a result, there is a significant influx of monocytes and T and B lymphocytes at the site of the lesion, which amplifies the inflammation and accelerates the development of atherosclerotic plaques.^101^ Therefore, focusing the treatment of atherosclerosis on enhancing immunosuppressive effects and expanding the number of Tregs could lead to substantial improvements (Table 2).

**TABLE 2:** Targeting Tregs for the prevention and treatment of cardiovascular diseases (CVDs).

Approach	Example	Mechanism	References
Antibodies and cytokines	IL‐37	Enhancement of Tregs inhibitory function and anti‐inflammatory cytokine secretion	[175]
	IL‐2	Tregs expansion	[176]
	CD3 antibody	Tregs‐associated cytokine production	[177, 178]
Vaccine	ox‐LDL‐ or ApoB100‐derived peptides and heat shock protein‐60	Tregs expansion, effector T‐cell suppression, and reduced atherosclerosis	[179, 180]
Adoptive transfer of regulatory T cells	Infusions of ex vivo expanded polyclonally activated Tregs	Increase in circulating polyclonal Tregs prevents the progression of atherosclerosis and induces a shift in plaque composition toward a more stable phenotype	[181, 182]
Physical therapy	Exposure to broadband ultraviolet B (spanning from 280 to 320 nm with a peak close to 313 nm)	Increase in Tregs number. Decrease in pro‐inflammatory IFN‐γ	[183]

Abbreviations: CD, clusters of differentiation; IFN‐γ, interferon‐gamma; IL, interleukin; ox‐LDL, oxidized of low‐density lipoprotein.

The Canakinumab Anti‐inflammatory Thrombosis Outcomes Study trial provided the first clinical evidence substantiating the correlation between inflammation and atherosclerotic disease. The study demonstrated that employing a fully human monoclonal antibody targeting IL‐1β for treatment resulted in diminished CVD risk among patients with previous MI.^184^ Two other recent clinical trials, the Colchicine Cardiovascular Outcomes Trial and Low‐dose Colchicine trial, have provided further evidence that treatment with colchicine, an anti‐inflammatory medication used for gout, familial Mediterranean fever, and pericarditis, significantly reduces the risk of cardiovascular events in patients with recent myocardial infarction or chronic coronary disease.^185^, ^186^, ^187^ These clinical trials support the efficacy of anti‐inflammatory therapies as a potential approach to the treatment of CVDs, particularly in patients with CVDs.

### 5.1 Induced expansion of Tregs

Clinical studies have shown that Tregs can be effectively expanded in vivo using approved drugs such as IL‐2 and rapamycin, resulting in significant clinical benefits.^188^ In humans, low doses of IL‐2 have been used to increase the number of natural Tregs without triggering problems related to IL‐2 signaling on natural killer cells or Teff. This method has consistently exhibited efficacy for Tregs expansion in patients with graft‐versus‐host disease, alopecia areata, type 1 diabetes, and hepatitis C virus‐induced vasculitis.^176^ CD4^+^Foxp3^+^ Tregs highly express CD25, a component of the high‐affinity IL‐2 receptor, and undergo active proliferation upon exposure to IL‐2.^82^ IL‐2 complexes, which are formed by recombinant mouse IL‐2 and anti‐IL‐2 monoclonal antibodies, efficiently expand CD4^+^CD25^+^Foxp3^+^ Tregs and reduce the impact of atherosclerosis in Ldlr^−/−^ or ApoE^−/−^ mice.^82^, ^118^ In addition, a study conducted by Tian and colleagues evaluated the safety and efficacy of low‐dose IL‐2 in patients with ischemic heart disease. The study showed that low‐dose IL‐2 successfully increased Tregs levels without significant adverse events. This finding suggests that low‐dose IL‐2 may have potential as an anti‐inflammatory therapy for the treatment of ischemic heart disease. However, larger studies are needed to confirm its safety and efficacy.^189^ As mentioned above, atherosclerosis can be considered an autoimmune disease triggered by the immune system's response to self‐proteins such as ox‐LDL or ApoB and heat shock protein‐60.^190^ Administration of ox‐LDL or ApoB100‐derived peptides and heat shock protein‐60 to ApoE^−/−^ or LDLr^−/−^ mice resulted in a significant decrease in the development of atherosclerosis, whether they were administered orally, nasally, or subcutaneously.^179^ This was associated with enhanced production of TGFβ and IL‐10 during antigen‐specific re‐exposure, as well as an increased number of Tregs.^179^, ^180^ Administering ApoB‐derived peptides subcutaneously in ApoE^−/−^ mice leads to Tregs expansion, Teff cell suppression, and reduced atherosclerosis.^191^ Likewise, oral administration of ox‐LDL, as well as heat shock protein‐60, increases CD4^+^CD25^+^Foxp3 Treg cells and reduces atherosclerosis in Ldlr^−/−^ mice.^192^, ^193^ Moreover, transfer of tolerogenic DCs pulsed with human ApoB‐100 and IL‐10 reduces atherosclerosis in transgenic Ldlr^−/−^ mice expressing human ApoB.^194^ These findings suggest that modulating the number of Tregs may be a potent therapeutic strategy for the treatment of patients with CVD. The use of antigen‐specific Tregs may be effective in the treatment of atherosclerosis. These approaches will likely be available in the near future.

### 5.2 Adoptive transfer of Tregs

As mentioned previously, a decrease in Tregs triggers the progression of atherosclerotic CVDs. Adoptive transfer of Tregs has been shown to prevent the development of atherosclerosis and induce a shift in plaque composition toward a more stable phenotype.^181^ This involves either isolating Tregs that have differentiated in vivo and expanding them ex vivo, or generating iTregs cells in vitro, and then transferring the Tregs/iTregs into the body.^195^ The potential prevention of atherosclerosis may be achieved by the adoptive transfer of CD4^+^CD25^+^ Tregs into ApoE^−/−^ mice.^182^ Additionally, the therapeutic benefits of transferring Tregs into patients have been widely acknowledged. The adoptive transfer of ex vivo expanded CD4^+^CD25^+^CD127^−^ Tregs has already shown promise in treating Graft‐versus‐host disease.^196^ The understanding of the function of Tregs in atherosclerosis is crucial for treatment considerations due to their safety and feasibility as a potential therapy. In addition, there is limited information to assess how long the effects of transferred Tregs will last. More data from these ongoing studies will be needed to inform the design of trials for patients with atherosclerotic disease.

### 5.3 Targeting Tregs with cytokines and antibodies

IL‐37 has been shown to play a promising role in atherosclerotic conditions as a potent inflammation inhibitor. It achieves this by rebalancing cytokine levels, which reduces excessive inflammation.^197^ Supplementation with exogenous IL‐37 increases the release of the anti‐inflammatory cytokines IL‐10 and TGF‐β from Tregs in vitro; both these cytokines are known to be released by Tregs. These results suggest that recombinant IL‐37 may play a role in mediating anti‐inflammatory functions in the atherosclerotic process, potentially by enhancing Tregs inhibitory function and anti‐inflammatory cytokine secretion.^175^ Intravenous administration of anti‐CD3‐specific antibodies has been shown to be effective in suppressing Teff immune responses and attenuating the development of atherosclerosis in mice, autoimmune diabetes in mice and humans, and acute transplant rejection in humans through induction of TGF‐β‐producing CD4^+^CD25^+^ Tregs.^177^, ^178^, ^198^ Administering anti‐CD3 antibodies orally or nasally can stimulate the production of CD4^+^LAP^+^ Tregs. CD4^+^LAP^+^ Tregs have been shown to inhibit experimental autoimmune conditions through a process driven by TGF‐β.^199^, ^200^ Additionally, treatment with anti‐CD3 antibodies combined with IL‐2 complexes successfully increased the number of Tregs and halted the progression of atherosclerosis in ApoE^−/−^ mice.^201^ Furthermore, physical therapy may serve as a preventive against atherosclerosis; exposure to broadband ultraviolet B (spanning from 280 to 320 nm with a peak close to 313 nm) reduces the onset and progression of atherosclerosis in ApoE^−/−^ mice. This is linked to an increase in CD4^+^Foxp3^+^ Tregs that exhibit enhanced suppressive abilities systemically, along with a decrease in pro‐inflammatory IFN‐γ generation from the T cells.^183^

### 5.4 Targeting Tregs function through a pharmacological approach

Telmisartan is an angiotensin II receptor antagonist that has demonstrated clinical effectiveness in patients with varying degrees of hypertension.^202^ The combination of telmisartan and rosuvastatin has been shown to simultaneously decrease plasma levels of TNF‐α and IL‐1β and increase Tregs cell frequency and FoxP3 mRNA expression in patients with hypertension.^203^ A recent study showed that the anti‐hypertensive amlodipine, in combination with atorvastatin, can improve the Th17/Tregs imbalance and ameliorate vascular endothelial function in patients with hypertension and atherosclerosis.^204^ In addition, indole propionic acid from the bacterial metabolism of tryptophan has been shown to have an inhibitory effect on fibrosis and inflammation at both the cellular and tissue levels.^205^, ^206^ The dietary supplementation of indole propionic acid has been reported to reduce blood pressure in a mouse model of high salt diet‐induced hypertension by decreasing pro‐inflammatory Th17 cells in the kidney, increasing anti‐inflammatory Tregs, and increasing urinary sodium excretion.^207^ It has been reported that lipid‐lowering drug statins can provide added athero‐protective benefits by increasing IL‐10 and TGF‐β levels while decreasing IFN‐γ levels.^208^ Atorvastatin and pravastatin have also been shown to increase the number of CD4^+^CD25^+^ Tregs in humans.^209^ A recent study showed that aspirin may attenuate the development of atherosclerosis by restoring the balance of the Th17‐Tregs axis and the Treg‐CD39‐CD73‐adenosine signaling pathways in ApoE^−/−^ mice.^83^ On the contrary, mycophenolate mofetil reduces the activation of DCs, macrophages, NKs, and T cells, enhances the levels of Tregs, decreases the generation of matrix metalloproteinase and cathepsins, and increases the expression of genes involved in cholesterol metabolism, such as ApoE, peroxisome proliferator activated receptor‐γ, and adenosine triphosphate‐binding cassette transporter‐A1/G1.^210^, ^211^ The activation of peroxisome proliferator activated receptor‐γ results in recruitment of Tregs, which aids in suppressing the inflammatory response of the vascular wall and slowing down the progression of atherosclerosis.^212^ Moreover, pioglitazone, an agonist of peroxisome proliferator activated receptor‐γ, improves atherosclerosis by regulating Th1/Th2 cell balance, enhancing Tregs responses, and increasing the levels of SMCs and collagen components in ApoE‐deficient mice.^212^, ^213^ Similarly, administration of low‐dose oral FTY720 (fingolimod), a sphingosine‐1‐phosphate receptor agonist, to ApoE^−/−^ mice fed a high‐cholesterol diet was found to contribute significantly to inhibition of the early development of atherosclerosis via induction of Tregs, and inhibition of the Teff cell response.^214^

Rapamycin, a mammalian target of rapamycin inhibitor, has been found to have immunosuppressive properties, promoting Tregs expansion while reducing Teff cell numbers. This suggests its potential application as a novel immunotherapy for T cell‐mediated CVD.^215^ Amygdalin, also known as vitamin B17, induces Tregs expansion and upregulates the expression of IL‐10 and TGF‐β in ApoE^−/−^ mice.^216^ Orally administered vitamin D3 protects against atherosclerosis by increasing Tregs numbers, promoting tolerogenic DCs, increasing IL‐10 expression, and decreasing IL‐12 expression.^217^, ^218^ Therefore, the manipulation of Tregs numbers and immunosuppressive functions through pharmacological approaches (Table 3) may be a promising therapeutic tool for treating atherosclerotic diseases.

**TABLE 3:** Pharmacological interventions on Tregs in cardiovascular disease (CVD).

Example	Mechanism	References
Mycophenolate mofetil	Inhibition of macrophages, DCs, T cells and NKs and increase in the number of Tregs	[210, 211]
Rapamycin	Expansion of Tregs and depletion of T effector cells	[215]
Fingolimod (FTY720)	Induction of regulatory T cells	[214]
Pioglitazone	Regulation of the balance between Teff and Tregs	[212, 213]
Aspirin	Restoring the balance of the Th17‐Tregs axis and the Tregs‐CD39‐CD73‐adenosine signaling pathways in ApoE^−/−^ mice	[83]
Statins Amlodipine and Atorvastatin Simvastatin	Increase in IL‐10 and TGF‐β levels and decrease in IFN‐γ levels Improvement of the Th17/Tregs imbalance and amelioration of vascular endothelial function Promote Tregs	[204, 208] [174]
Vitamin D3	Induction of Tregs and tolerogenic DCs	[217, 218]
Amygdalin (vitamin B17) Indole propionic acid	Tregs expansion and upregulation of the expression of IL‐10 and TGF‐β Decrease in pro‐inflammatory Th17 cells in the kidney, and increases in anti‐inflammatory Tregs and urinary sodium excretion	[216] [207]

Abbreviations: DC, dendritic cell; IFN‐γ‐interferon‐gamma; IL, interleukin; NK, natural killer; TGF‐β, transforming growth factor‐beta; Th, T helper.

## 6 CONCLUSIONS

The burden of hypertension and CVDs continues to increase worldwide. However, additional evidence is needed from numerous randomized controlled clinical trials regarding the importance of Tregs in the treatment of these diseases. Some clinical trials have focused on determining the number, proportion, and function of Tregs as a biomarker of disease severity in CVD patients. However, before their results can be used in clinical trials to improve hypertension and CVD, extensive basic experimental researches and stringent efficacy and safety evaluations are required, whether for the addition of endogenous Tregs or the injection of exogenous Tregs to enhance their function. This review delivers a summary of the clinical studies and basic experimental trials conducted on targeted Tregs for hypertension and CVDs and serves as a foundation for future Tregs research. These breakthroughs are expected not only to tackle present challenges but also to pave the way for innovative therapeutic options in the near future. Collectively, they have the potential to revolutionize the approach toward hypertension and atherosclerosis treatment.

## AUTHOR CONTRIBUTIONS

Vipin Kumar contributed to the conceptualization, investigation, and writing—original draft. Megumi Narisawa drew the figures and contributed to the writing—review and editing. Xian Wu Cheng revised and critically reviewed the article. All authors have read and agreed to the published version of the manuscript.

## FUNDING INFORMATION

This work was supported in part by grants from the National Natural Science Foundation of China (nos.: 81770485 and 82370424 to X.W.C.).

## DISCLOSURES

The authors declare no potential conflicts of interest with respect to the research, authorship, and/or publication of this manuscript.

## Data Availability

None.
